# Design and Synthesis of Bicyclo[4.3.0]nonene Nucleoside Analogues

**DOI:** 10.1021/acs.orglett.3c03590

**Published:** 2023-12-05

**Authors:** Stephan Scheeff, Yan Wang, Mao-Yun Lyu, Behzad Nasiri Ahmadabadi, Sam Chun Kit Hau, Tony K. C. Hui, Yufeng Zhang, Zhong Zuo, Renee Wan Yi Chan, Billy Wai-Lung Ng

**Affiliations:** a School of Pharmacy, Faculty of Medicine, The Chinese University of Hong Kong, Shatin , Hong Kong; b Department of Paediatrics, Faculty of Medicine, The Chinese University of Hong Kong, Shatin , Hong Kong; c Department of Chemistry, The Chinese University of Hong Kong, Shatin , Hong Kong; d Primemax Biotech Ltd., Shatin , Hong Kong; e Li Ka Shing Institute of Health Sciences, Faculty of Medicine, The Chinese University of Hong Kong, Shatin , Hong Kong; f Hong Kong Hub of Paediatric Excellence, The Chinese University of Hong Kong, Kowloon Bay, Hong Kong; g S.H. Ho Research Centre for Infectious Diseases, Faculty of Medicine, The Chinese University of Hong Kong, Shatin, Hong Kong

## Abstract

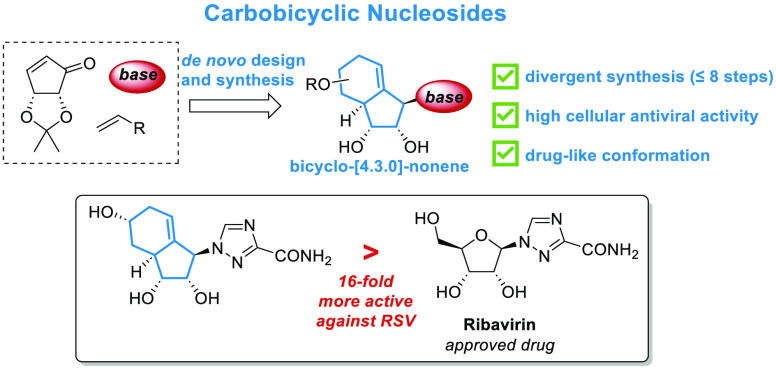

Nucleoside analogues are effective antiviral agents, and the continuous emergence of pathogenic viruses demands the development of novel and structurally diverse analogues. Here, we present the design and synthesis of novel nucleoside analogues with a carbobicyclic core, which mimics the conformation of natural ribonucleosides. Employing a divergent synthetic route featuring an intermolecular Diels–Alder reaction, we successfully synthesized carbobicyclic nucleoside analogues with high antiviral efficacy against respiratory syncytial virus.

Nucleoside analogues (NAs) are effective antiviral drugs and are used to treat many pathogenic viral infections.^1^ The continuous emergence of new viruses requires the development of novel antivirals, and structural diversity is key to overcoming drug resistance and enhancing combination therapies. Herein, we report the design and synthesis of NAs with a bicyclo[4.3.0]nonene carbobicyclic core [**IV** (Figure 1C)] as a novel template for antiviral NAs with activity against respiratory syncytial virus (RSV). While a traditional approach focuses on modifying the nucleobase, exemplified by the antivirals ribavirin [**Ia** (Figure 1A)]^2^ and molnupiravir (**Ib**),^3^ other strategies involve simplification, substitution, or modification of the ribose core (see Figure S1). Substitution of the ribose ring oxygen with a methylene group creates an important antiviral class, the carbocyclic nucleoside analogues (Figure 1B). However, these NAs are uncommon, as the absence of anomeric stabilization often leads to unnatural conformations and reduced biological activity.^4^ To address this, carbobicyclic analogues have been developed to lock the pseudosugar ring in its bioactive conformation. Notably, bicyclo[3.1.0]hexane analogue N-MCT [**IIb** (Figure 1B)]^5^ has shown superior drug-like properties^6^ and promising antiviral activities. Another strategy for generating a favorable conformation involves substituting the ribose ring oxygen^7^ with an exocyclic alkene, a modification exemplified by entecavir (**IIa**).^8^

**Figure 1:**
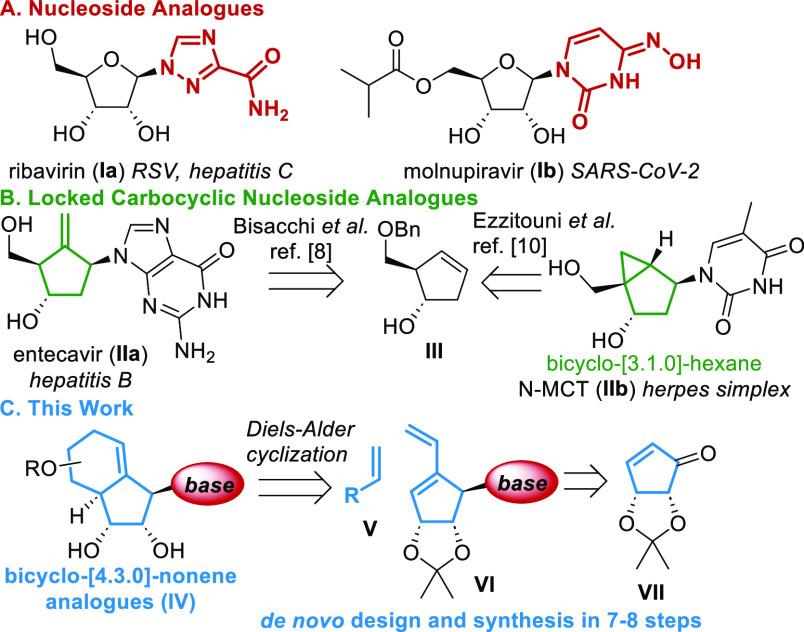
Design and retrosynthetic analysis of bicyclo[4.3.0]nonene structure type nucleoside analogues.

The synthesis of conformationally locked nucleosides presents notable challenges, primarily due to the necessity of designing them *ab initio* and their high density of stereochemical information.^9^ For instance, the synthesis of **IIa** can be achieved only through a complete total synthesis route in 8–15 synthetic steps.^8^ Similarly, N-MCT **IIb**, another locked carbocyclic analogue, is achieved after 11 steps from the same precursor **III**.^10^ As a result, the exploration of structure–activity relationships (SARs) in carbocylic NAs is often hindered.

The design of NAs that can act as alternatives to ribonucleosides while retaining bioactive conformations is challenging. As an example, saturated bicyclo[4.3.0]nonane NAs are candidates with the potential for functionalization and SAR studies. However, their application is compromised by their unfavorable conformation, lack of biological activity, and inefficient synthesis (16–19 steps as shown in Figure S2).^9c^ We envisioned that the incorporation of an endocyclic double bond into the unsaturated bicyclo[4.3.0]nonene scaffold [**IV** (Figure 1C)] could align these analogues conformationally with natural ribonucleosides. This modification not only simplifies the synthesis process but also potentially enhances the biological activity, addressing the limitations of previous designs. Building upon our expertise in carbohydrate chemistry,^11^ we have designed a concise and divergent route yielding a structurally distinct class of bicyclo[4.3.0]nonene NAs.

Initially, we launched the synthesis of carbobicyclic analogues of ribavirin. Ribavirin itself is a Food and Drug Administration-approved antiviral drug for treating infections caused by RSV. Our goal was to generate a stereo- and regiodiverse library of analogues, which facilitates the exploration of the SAR of our compounds. The stereodivergence is achieved by the Diels–Alder reaction between diene **VI** and dienophile **V**. Diene **VI** itself should be available from **VII** by the Mitsunobu reaction. The alternative reaction pathway in which the Diels–Alder reaction occurs before the Mitsunobu reaction was not successful.^9c^

We synthesized Diels–Alder precursor **6** from commercially available enone **2**(12) in four steps (see Scheme 1). First, α-iodination of enone **2** under a Baylis–Hillman type pathway followed by Luche reduction gave alcohol **3** stereospecifically.^13^ The reduction product could be employed in the Mitsunobu coupling with triazole **4** yielding **5a** and **5b** in 78% total yield as separable regioisomers.^2,14^ Notably, with THF, *iso*-ribavirin type isomer **5b** was afforded as the major product (**5a**:**5b** ratio of 1:4). While using DCM, both regioisomers were obtained in a nearly equimolar ratio, a phenomenon that had not previously been reported. Both isomers can be converted to the corresponding dienes **6a** and **6b** in excellent yield using a Stille cross-coupling.^15^

**Scheme 1:**
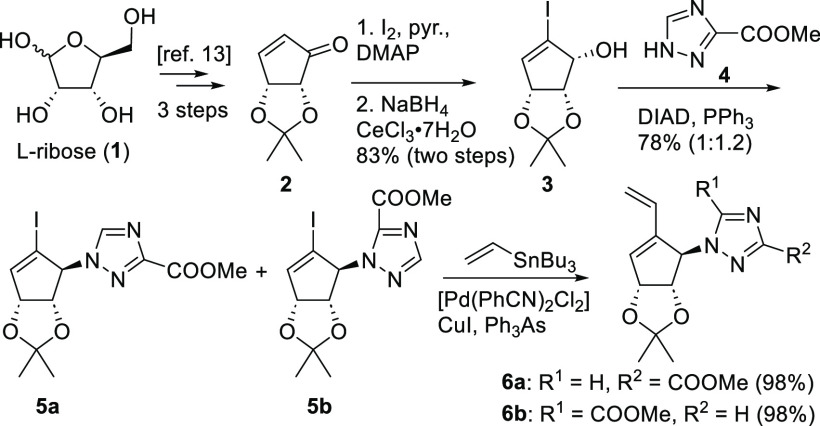
Synthesis of Diels–Alder Precursor **6** from **2**

In the intermolecular Diels–Alder reaction, an activated dienophile was necessary, and thus, vinyl boronate was used to allow the efficient installation of a pseudo-C5′-OH functionality (Scheme 2)^16^ through facile oxidative cleavage of the boronate with NaBO_3_. We envisioned that the dienophile approaches diene **6** from the upper side, avoiding the steric interactions with the isopropylidene acetal group. To our delight, the desired alcohol **7** was isolated as a single isomer. For the two other main isomers, separation was possible after esterification to **8** and **9** (for details, see Figure S3). Adducts **7**–**9** could be isolated in 67–70% total yield from **6**. The synthesis of the designed ribavirin analogues was then completed in two or three steps (see Table 1). First, the methyl ester of the triazole nucleobase was transformed into the amide,^3,14a−14c,17^ and then nucleoside analogues **10**–**12** were achieved by deprotection. In detail, ribavirin analogue **10a** and isomer **10b** were obtained by treatment of **7** with methanolic ammonia and subsequent acidic treatment (TFA in H_2_O/MeOH). An additional esterification step^18^ yielded C5′-OH esters **10c** and **10d**. In the case of **8** and **9**, amidation did not lead to cleavage of the *iso*-butyric acid esters (at pseudo-C6′), and analogues **11c**, **11d**, **12c**, and **12d** were isolated after acidic deprotection. Amidation with subsequent one-pot K_2_CO_3_-mediated ester cleavage, followed by acidic acetonide deprotection, gave **11a**, **11b**, **12a**, and **12b** in good yields. In short, a total of 12 ribavirin analogues were synthesized in seven or eight steps from commercially available enone **2**.

**Scheme 2:**
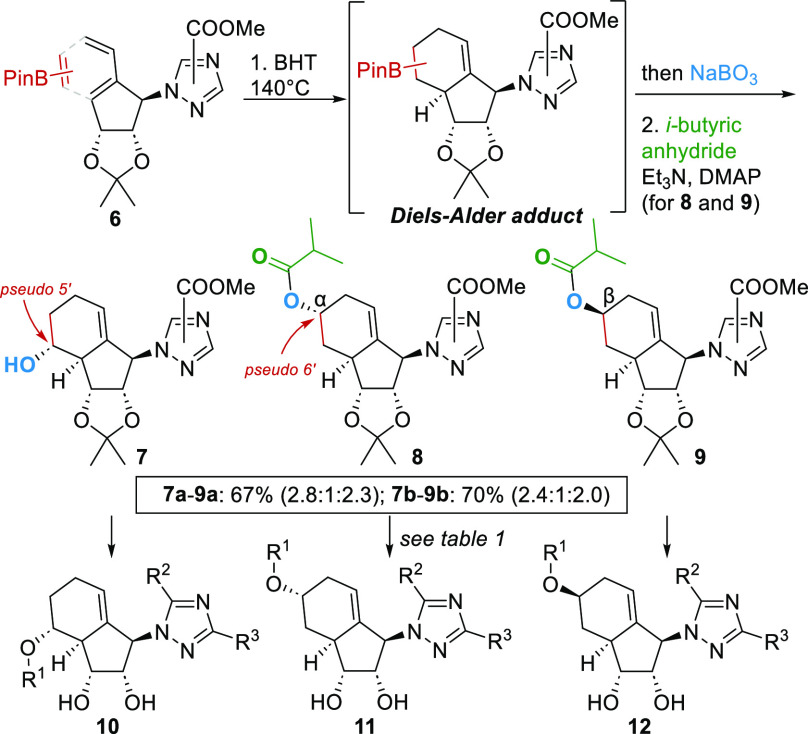
Diels–Alder Reaction toward **10**–**12**

**Table 1:**
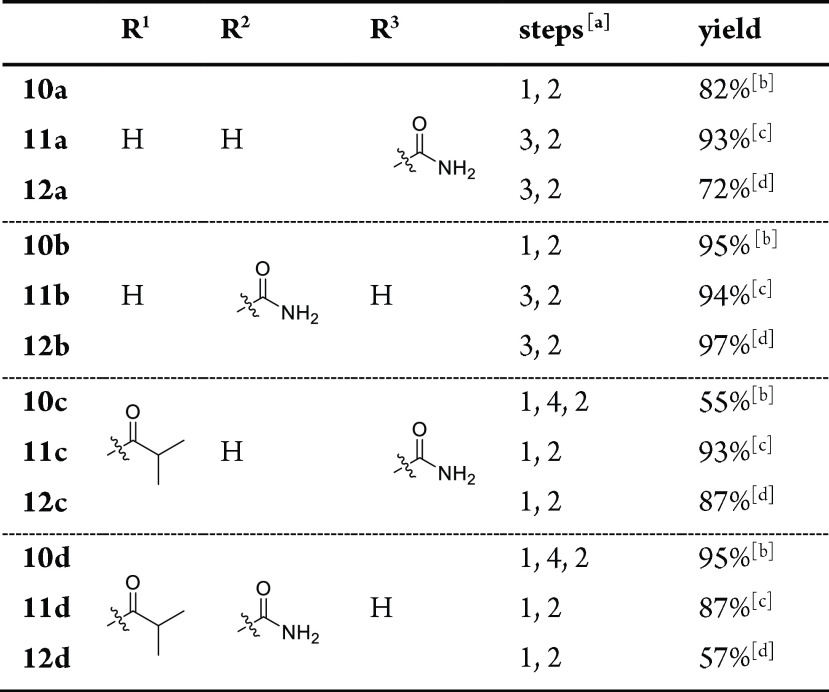
Synthesis of Compounds **10**–**12** from **7**–**9**, Respectively

a Conditions: (1) NH_3_, MeOH; (2) TFA; (3) NH_3_, MeOH, then K_2_CO_3_; (4) isobutyric anhydride, Et_3_N, DMAP.

b From **7**.

c From **8**.

d From **9**.

The relative configurations of isomers **10a**–**12a** among others (see the Supporting Information) were determined by two-dimensional NMR studies, and all other intermediates were assigned accordingly. This assessment is in accordance with the X-ray single-crystal analysis of **12b**, which validated our initial hypothesis that the bicyclic core might have a ribose-like conformation [*P* = +24.0°; C3′-endo (see Figure S4)]. This conformation is also observed in natural nucleosides and therefore is promising for biological activity.^4b^ Superposition of the three-dimensional X-ray structure of **12b** with the carbasugar entecavir^19^ revealed an identical conformation of the carbasugar core of both structures (Figure 2A). More importantly, the comparison with the authentic ribose-based ribavirin^20^ revealed a nearly identical core conformation albeit slightly twisted. Thus, the nucleoside analogues adopted a favorable drug-like conformation. This is in stark contrast to the case for the previously reported saturated bicyclo[4.3.0]nonane nucleoside analogues, which have a biologically inactive C1′-exo conformation.^9c^

**Figure 2:**
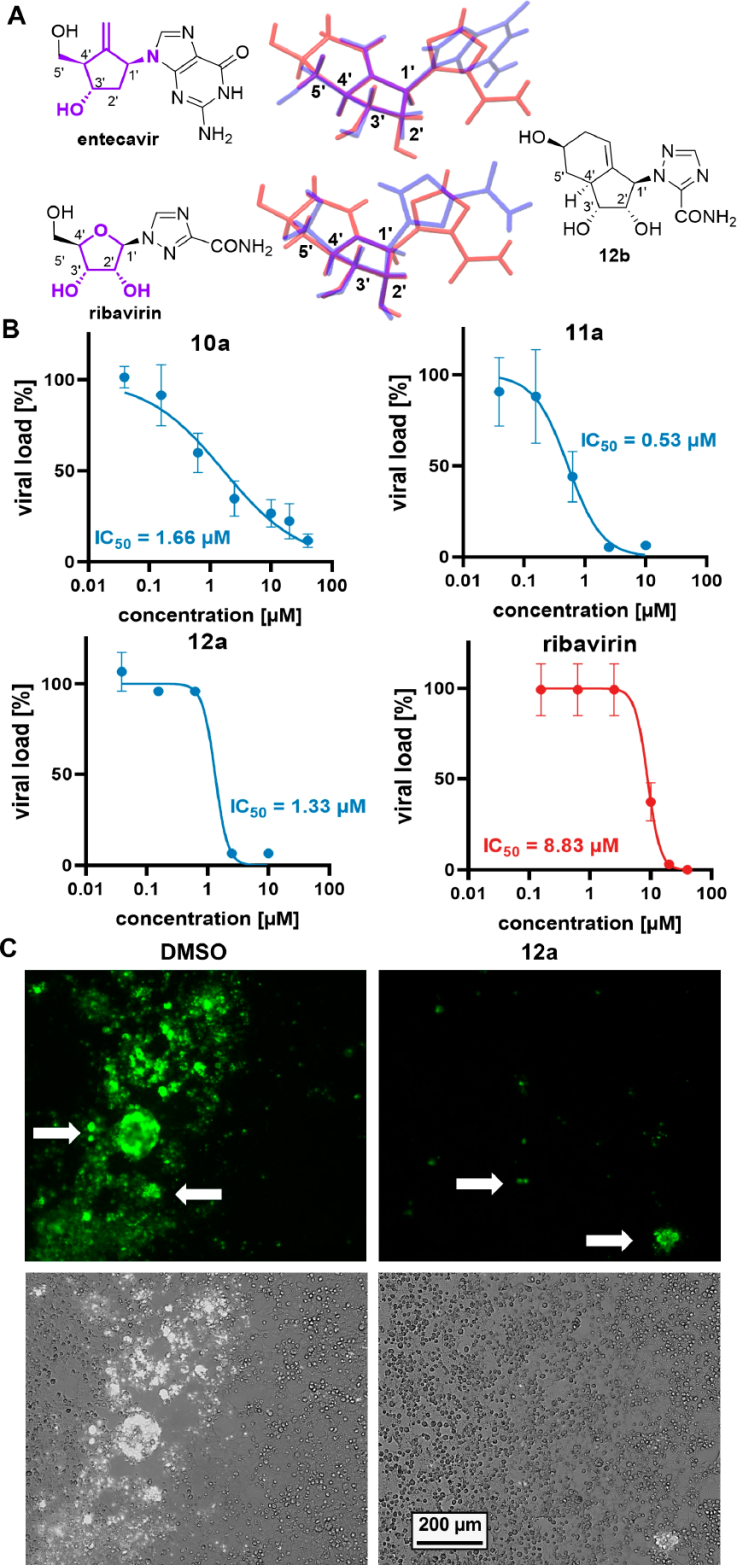
(A) Superposition of X-ray structures of entecavir or ribavirin (blue) with **12b** (red). (B) Antiviral activity (HEp-2 cells, RSV) of **10a–12a** (percentage ± the standard error of the mean of the DMSO control; *n* = 3). (C) Representative fluorescence images of immunostained RSV (white arrows) in infected HEp-2 cells (gray) with DMSO or **12a** treatment (2 μM).

We then assessed their effectiveness in inhibiting RSV replication by using an established model (RSV-A, HEp-2 cells).^21^ First, we tested parental compounds **10a**–**12a** and **10b–12b** at concentrations of 2 and 40 μM, respectively, and observed promising antiviral activities (Figures S5 and S6). The ribavirin analogues (**10a–12a**) compared to the *iso*-ribavirin (**10b–12b**) exhibited similar levels of activity. Additionally, analysis of the prodrug variants [**10c–12c** (see Figure S8)] revealed no significant difference in antiviral potency compared to that of their active form (**10a**–**12a**). Although there is no difference in activity, these subtle structural differences impact the pharmacokinetic properties. The *iso*-butyrate analogues demonstrated more favorable predicted values for intestinal permeability (*P*_app_), solubility (log *S*), lipophilicity (log *D*), and the partition coefficient (log *P*) compared to thoose of the non-ester analogues (see table S1). In addition, the predicted intestinal permeability of analogues **10**–**12** is higher than that of ribavirin, suggesting a potential for better oral bioavailability.^22^

We then followed up to determine the IC_50_ against RSV and the cytotoxicity (CC_50_). All three derivatives, **10a–12a** showed excellent anti-RSV activity with IC_50_ values in a low micromolar range [0.53–1.66 μM (Figure 2B)], even lower than that of ribavirin (IC_50_ = 8.83 μM). Analogue **11a** was the most active compound, with activity that was 16 times higher than that of ribavirin. It is noteworthy that all three derivatives have a CC_50_ value of >40 μM, indicating a high selectivity and thus a wide therapeutic window (see Figure S9). Immunostaining of RSV-infected cells validated the screening results (Figure 2C), where the treatment with **12a** (2 μM) leads to a significant inhibition (see also Figure S6).

To further explore the potential of the bicyclic nucleoside analogues fully, we were interested in whether the antiviral activity was limited to ribavirin type analogues. Therefore, we synthesized carbobicyclic uridine analogues **19**–**21** to rule out the possibility that the antiviral effect stemmed from the triazole base alone. Uridine analogues **19**–**21** were synthesized in analogy to the previous synthesis as shown in Scheme 3.

**Scheme 3:**
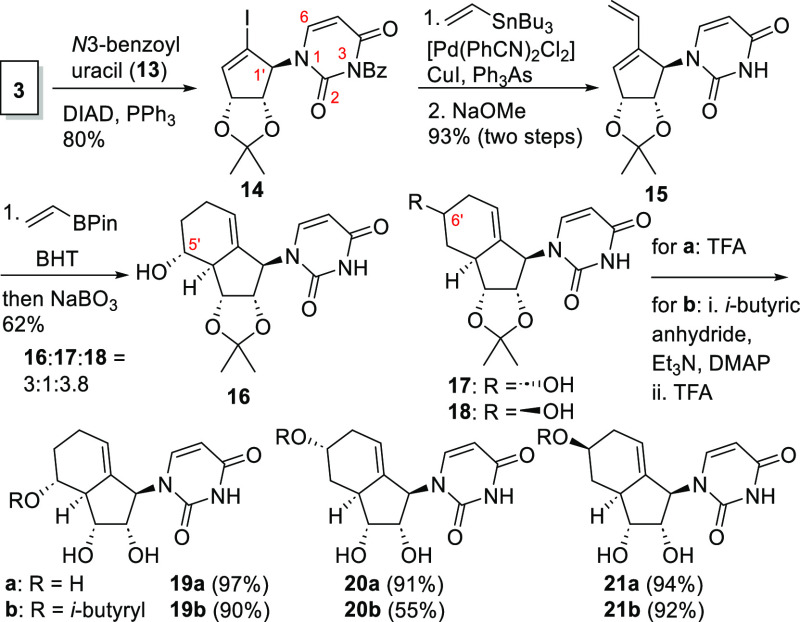
Synthesis of Uridine Analogues **19**–**21**

First, alcohol **3** was coupled with N3-Bz uracil (**13**)^23^ under Mitsunobu conditions. Only N1-coupled (vs O2) products were isolated.^23b,24^ Stille cross-coupling of **14** followed by debenzoylation gave an excellent yield of the desired Diels–Alder precursor **15**. In our pilot Diels–Alder reaction, the N3-benzoyl group was partially cleaved under the thermal conditions, leading to difficulties during the isolation of the Diels–Alder adduct. Thus, debenzoylation prior to the Diels–Alder reaction was essential. The conversion of uracil diene **15** was relatively retarded compared to that of its ribavirin analogue; the optimized molar ratio of the dienophile furnished diverse cycloadducts **16**–**18** in moderate yield with regio- and diastereoselectivity comparable to that of the ribavirin analogues. The final esterification and/or deprotection led to carbobicyclic uracil analogues **19**–**21** in seven or eight steps from **2**.

During NMR analysis of the uracil analogues, we observed significant peak broadening, suggesting that the analogues exist as two structural conformers at room temperature. Variable-temperature NMR studies showed that both conformers merge at increased temperatures [315 K (see Figure S10)]. The desired connectivity (N1 vs O2) was confirmed by NMR (characteristic ^1^H and ^13^C shift at C1′)^25^ and NOESY. In addition to NOE, the relative configuration was further confirmed in analogy with ribavirin type analogues, as the identical core structure (e.g., **10** and **19**) shares characteristic ^1^H NMR chemical shifts and splitting patterns (see Figures S11–S13).

In the subsequent cellular assays, uridine analogues **19a** and **21a** showed good antiviral activity against RSV and almost no cytotoxicity whereas **20a** at 40 μM showed only a nonsignificant reduction of viral load (see Figures S5 and S6). We demonstrate a dose-dependent inhibition for pseudo-C5′-OH analogue **19a** with an IC_50_ of 6.94 μM and low cytotoxicity [CC_50_ > 40 μM (Figure S9)]. Collectively, these data confirmed that the carbobicyclic core contributes to antiviral activity. Although our compounds are structural analogues of traditional nucleosides, their mechanisms of action may differ from those of the known nucleoside analogues. Further studies are essential to elucidate their antiviral mechanisms.

In summary, we report herein the design, synthesis, and biological activity of a new class of nucleoside analogues with a bicyclo[4.3.0]nonene core. The synthesized ribavirin type carbobicyclic analogues exhibit a ribose-like conformation and demonstrate promising antiviral activity with minimal cytotoxicity. The versatility of our synthetic approach will allow for further diversification through the incorporation of various nucleobases such as adenosine, guanosine, and 5-fluorouracil. Thus, this new antiviral structural motif opens opportunities to combat emerging and future outbreaks of viral diseases and antiviral drug resistance.

## Data Availability

The data underlying this study are available in the published article and its Supporting Information.

## Supporting Information Available

Additional figures, experimental data, characterization data, NMR spectra, and crystallographic data (PDF)

## Dedication

Dedicated to Professor Tony K. M. Shing.
